# Astroglia in the Vulnerability to and Maintenance of Stress-Mediated Neuropathology and Depression

**DOI:** 10.3389/fncel.2022.869779

**Published:** 2022-04-22

**Authors:** José Javier Miguel-Hidalgo

**Affiliations:** Department of Psychiatry and Human Behavior, University of Mississippi Medical Center, Jackson, MS, United States

**Keywords:** astrocytes, stress, depression, pathology, epigenetics, glutamate

## Abstract

Significant stress exposure and psychiatric depression are associated with morphological, biochemical, and physiological disturbances of astrocytes in specific brain regions relevant to the pathophysiology of those disorders, suggesting that astrocytes are involved in the mechanisms underlying the vulnerability to or maintenance of stress-related neuropathology and depression. To understand those mechanisms a variety of studies have probed the effect of various modalities of stress exposure on the metabolism, gene expression and plasticity of astrocytes. These studies have uncovered the participation of various cellular pathways, such as those for intracellular calcium regulation, neuroimmune responses, extracellular ionic regulation, gap junctions-based cellular communication, and regulation of neurotransmitter and gliotransmitter release and uptake. More recently epigenetic modifications resulting from exposure to chronic forms of stress or to early life adversity have been suggested to affect not only neuronal mechanisms but also gene expression and physiology of astrocytes and other glial cells. However, much remains to be learned to understand the specific role of those and other modifications in the astroglial contribution to the vulnerability to and maintenance of stress-related disorders and depression, and for leveraging that knowledge to achieve more effective psychiatric therapies.

## Introduction

Stress and the systemic and neurophysiological responses it triggers are major risk factors for depression and other psychiatric disorders. In these disorders as well as in animal models for the effects of stress, astrocytes in several brain regions display morphological and molecular alterations (Bender et al., 2016; Murphy-Royal et al., 2019) that may result in grave consequences for the physiology and connectivity of brain circuits (Fields et al., 2015). A variety of chronic stress-inducing procedures in animals, such as chronic unpredictable stress, repeated fearful social interactions or recurrent restraint in rodents result in reductions in the numbers of astrocyte labeled for specific markers such as glial fibrillary acidic proteins (GFAP), in the levels the mRNA expression of the markers, and in the extent of the labeled processes that stem from their cell bodies in prefrontal cortical areas, the hippocampus or other brain regions (Banasr and Duman, 2008; Liu et al., 2009; Banasr et al., 2010; Araya-Callis et al., 2012; Imbe et al., 2012, 2013; Li L. F. et al., 2013; Li Y. S. et al., 2013; Tynan et al., 2013; Codeluppi et al., 2021). Interestingly, laboratory rats of the Wistar Kyoto strain, known to display spontaneously some depression-like behaviors, show comparable lower levels of GFAP astrocyte immunostaining (Gosselin et al., 2009). Social defeat stress in a different species, the tree shrew, a marsupial mammal, leads to significant decrease in GFAP positive astrocyte and their cell body size in the hippocampus (Czeh et al., 2006).

Although most examples of chronic stress result in reduction of astrocytes’ cell size or their GFAP expression as well as reduced levels of glutamate transporters, acute or short exposures to various stress procedures appear to augment rather than decrease markers of astrocytes (Bender et al., 2016; Yoshino et al., 2020). For example, two exposures in two consecutive days to electric shocks produced learned hopelessness in rats, but they increased the expression of glutamate transporter 1 (GLT-1) and glutamine synthetase (Yoshino et al., 2020). Similar results in other studies of the effects of short exposure to stress or adversity, have led some researchers to suggest that moderate or short stress [with the exception perhaps of post-traumatic stress disorder (PTSD)] rather than contributing to eventual depression would increase resilience and that astrocytes alterations may also be involved in positive effects of short-term stresses (Franklin et al., 2012; Menard et al., 2017). Nonetheless, most of the examples of astrocyte involvement in stress effects and depression-related behaviors discussed below are mostly related to chronic stress or stress undergone in early life stages.

It must also be pointed out that repeated exposure to altered concentrations of glucocorticoids in chronic stress may be a major factor determining the pathological changes in astrocytes that contribute to depression-like behavior. For instance, chronic treatment with corticosterone in mice results in reduced numbers of GFAP-positive astrocytes, smaller volume of their cells bodies and shorter process length and induces hippocampal atrophy in mice (Zhang et al., 2015). More recently, exposure to chronic social defeat stress, lipopolysaccharide or corticosteroid analog dexamethasone (DEX) have been shown to result in marked downregulation of glucocorticoid receptor (GR) expression in astrocytes and much less so in neurons in mice (Lu et al., 2021). Absence of those receptors in astrocytes results in depression-like behaviors while astrocyte-specific restoration of the receptors in the medial prefrontal cortex greatly mitigated the depression-like behavior (Lu et al., 2021). Thus, depletion of GR expression in astrocytes of the prefrontal cortex and other brain regions may be a major factor in the various astrocyte-specific molecular changes and the mechanism by which they contribute to long lasting behavioral and emotional effects of stress.

## Astrocytes and Glutamate Neurotranmission and Reuptake

### Stress and the Role of NMDA-Type Glutamate Receptors

In animal models, stress that results in depression- and anxiety-like behaviors induces profound changes in various components of glutamate- and gamma-aminobutyric acid (GABA)-driven neurotransmission (Choudary et al., 2005; Kendell et al., 2005; Karolewicz et al., 2010; McKlveen et al., 2016; Réus et al., 2016). At the neuronal level, chronic stress and excess glucocorticoids lead to decreased expression of glutamate receptors in the prefrontal cortex, an effect that is dependent on the activation of glucocorticoid receptors (Yuen et al., 2012). In human subjects, elevated levels for N-methyl-D-aspartate-type (NMDA) and metabotropic glutamate receptor subunits were measured in the locus coeruleus of depressed subjects but not in pyramidal cells of the prefrontal cortex (Chandley et al., 2014), although a study found increases of metabotropic glutamate receptor 2/3 in Brodmann’s area 10 of the prefrontal cortex (Feyissa et al., 2010). However, the regulation of glutamatergic neurotransmission is also critically dependent on the ability to reuptake released glutamate and recycle it to glutamine by astrocyte processes surrounding synapses, glutamine then being returned to the presynaptic element for further generation and release of glutamate. Thus, astrocytes have a critical role not only in terminating synaptic actions of glutamate but also in ensuring the continuous supply of releasable glutamate (Had-Aissouni et al., 2002).

### Astrocytic Glutamate Transporters and Glutamine Synthetase in the Vulnerability to Stress and Depression

Regulation of the extracellular content of glutamate at synaptic and extra-synaptic sites is highly dependent on the activity of glutamate transporters EAAT1 and EAAT2 in the membranes of astrocytes processes, while the recycling of glutamate to glutamine within astrocytes is also performed in astrocytes by the action of glutamine synthetase. In human subjects with depression there are lower levels of glutamate transporters in the prefrontal cortex (Miguel-Hidalgo et al., 2010; Nagy et al., 2015). Similarly, in rodents subjected to chronic stress and in other animal models exhibiting depressive behaviors there is also a decrease in the expression of astrocytic glutamate transporters (Zink et al., 2010; Gomez-Galan et al., 2013; Piao et al., 2019) or glutamate reuptake (Almeida et al., 2010; de Vasconcellos-Bittencourt et al., 2011) in the cortex and hippocampus as compared to non-stressed controls.

Reduction in astrocytic transport relevant to the manifestation of depressive behaviors is not limited to cortical regions of the brain but extends as well to subcortical structures that have been long known to substantially contribute to the pathophysiology of stress effects and depression. In mice with depression-like behaviors caused by early life stress, the immunostaining of GLT1 astrocyte transporters and the thickness of astrocyte processes are reduced in the corticotrophin-releasing factor (CRF)-expressing dorsal-medial (MPD) neurons of the hypothalamus, effects which are concomitant with increased excitability of the surrounding neurons (Gunn et al., 2013). Also in mice, decreased activity of GLT1 transporters in the habenula promotes depression-like behaviors and increases the behavioral vulnerability to depression-like behavioral effects of chronic stress (Cui et al., 2014). Loss of astrocyte glutamate transporter GLT-1 was observed also in the periaqueductal gray matter of rats subjected to chronic restraint stress (Imbe et al., 2012). Thus, the available evidence strongly suggests that prolonged or repetitive stress results in a generalized or, at least, amply extensive decrease in the astrocytic cellular systems for glutamate reuptake, and that behavioral effects may be greatly influenced by the corresponding disturbances in neuronal excitability. This involvement of glutamate reuptake dysfunction in the depression-like behavioral effects of stress is supported also by the demonstration that administration of riluzole, a compound that stimulates the reuptake of glutamate by astrocytes, and also reduces its release, mitigates depression-like behaviors and prevents alterations of astrocyte metabolism and GFAP mRNA expression in rodent models of stress (Banasr et al., 2010). Likewise, ceftriaxone, a beta-lactam antibiotic that favors glutamate uptake, results in antidepressant-like effects across models of stress in mice (Mineur et al., 2007).

### Gap Junctions of Astrocytes in Stress and Depression

In animals under chronic stress and in humans with major depression) there are drastically low levels of astrocytic gap junction proteins connexin 43 (Cx43) and connexin 30 (Cx30) in the prefrontal cortex (PFC) as compared to controls (Ernst et al., 2011; Sun et al., 2012; Miguel-Hidalgo et al., 2014, 2018; Nagy et al., 2016). Likewise, some animal models of stress leading to depression-like behaviors have demonstrated a region-specific decrease in myelination markers after prolonged stress, partially reversible with antidepressant treatments or myelin-promoting agents (Liu et al., 2012, 2016). This is consistent with findings in the PFC of human subjects with depression, where there is reduced expression of myelin- and astrocyte-related mRNAs (Liu et al., 2012, 2016; Rajkowska et al., 2015; Miguel-Hidalgo et al., 2017, 2018). More recently, highly suggestive evidence of reduced gap junction coupling between astrocytes and oligodendrocytes has been also observed in the anterior cingulate cortex of subjects with depression (Tanti et al., 2019). Gap junctions formed by astrocyte connexons (made of Cx43 or Cx30 subunits) interacting with the corresponding connexons from oligodendrocytes (made of Cx47 or C32) (Giaume and McCarthy, 1996) have proven crucial for the maintenance of myelin, as mice with KO for different combinations of astrocyte and oligodendrocyte connexins demonstrate variable degrees of myelin vacuolization and degradation, and profound behavioral deficits (Lutz et al., 2009; Magnotti et al., 2011; Li et al., 2014; Basu and Sarma, 2018). Whether chronic stress or sustained high corticosteroids in the blood circulation or the brain results in deficient myelin formation or maintenance mediated by reduced astrocyte connexins is less well established, although recently published evidence from our laboratory, has provided the first proof-of-concept evidence that stress, or high corticosterone during myelination, results in concomitant myelin and Cx43/Cx30 alterations that involve lower immunoreactivity for myelin basic protein and those connexins *in vivo* (Miguel-Hidalgo et al., 2018) and *in vitro* (Miguel-Hidalgo et al., 2019), and decreased morphological indices of myelination *in vitro*. We also have observed that concurrent reductions of connexins and myelin proteins in cortical primary cell cultures *in vitro* can both be prevented by the glucocorticoid receptor antagonist mifepristone (Miguel-Hidalgo et al., 2019).

Mechanisms by which glucocorticoids regulate the expression, turnover or function of Cx43-containing gap junctions have been partially characterized in cells from tissues outside of nervous system. In cells from these tissues corticosterone (CORT) causes Cx43 degradation through autophagy mechanisms (Gao et al., 2016) or the activation of the Akt/mTOR signaling pathway (Shen et al., 2016). Activation of GC receptors is involved in those mechanisms because antagonism with mifepristone blocks CORT effects. In addition, some *in vitro* studies with primary cortical astrocytes, and *in vivo* research in the cerebral cortex, have found that high CORT levels cause reduction in Cx43 protein (Xia et al., 2017, 2018) or mRNA (Samarasinghe et al., 2012; Carter et al., 2013) and that inactivation of p38 and c-fos/AP1 pathways (Morioka et al., 2014) may also lead to reduced Cx43. Also *in vivo*, some antidepressants, or GC receptor antagonists such as mifepristone mitigate the reduction of Cx43 caused by chronic unpredictable stress in animals (Sun et al., 2012; Xia et al., 2017). However, the mechanisms by which glucocorticoid receptor activation leads to Cx43 reductions in the CNS, and particularly in the PFC in stress or in postmortem brains from subjects with depression, remain to be ascertained. Neurodevelopmental research in prenatal neural progenitors has also indicated that the inactivation and reduction Cx43 gap junctions can be mediated by non-genomic actions of caveolin-1-attached GC receptors through the activation of the Raf-RAS-ERK1/2 pathway (Pedram et al., 2006; Hammes and Levin, 2007; Samarasinghe et al., 2012), although other effects on astrocytes may depend on the genomic pathway of GR activation (Kumar et al., 1986). Furthermore caveolin-1 is strongly expressed in astrocytes (Yun et al., 2011). Excess CORT-related decreases of brain-derived neurotrophic factor (BDNF) may also contribute to the reduction of Cx43, as shown in hippocampal astrocytes although this mechanism might not operate in the PFC (Schaaf et al., 1998). Thus, increased autophagy, AKt/mTOR activation, ERK-activation/Cx43-reduction by non-genomic GR mechanisms, or excess GC-induced BDNF decrease may also lead to alterations in the structure and function of astrocyte gap junctions. However, it is unknown whether and which of those mechanisms operates in the effects of stress (or high CORT) on astrocyte connexins or their companion oligodendrocyte connexins in the PFC. Interestingly, in our recent *in vitro* studies of mixed rat cortical primary cell cultures, we observed that reduction of Cx43 with a high dose of CORT analog DEX, which has a very high effectivity for genomic GR actions, was noticeably smaller than with high CORT (Miguel-Hidalgo et al., 2019), suggesting that, besides the genome-acting GC receptors, reductions of Cx43 with stress may be substantially due to non-genomic effects of GR activation.

An important contribution of astrocyte Cx43 and Cx30 to the maintenance of myelin integrity has been established in rodent models with KO of Cx43 and Cx30 expression alone or in combination with their KO of Cx47 and Cx32 oligodendrocyte partners, where the KO leads in many instances to patent myelin disorganization (Lutz et al., 2009; Magnotti et al., 2011; Tress et al., 2012; Li et al., 2014). However, it is yet unknown whether connexin decreases caused by high glucocorticoids or stress is part of mechanisms that would lead to reduced expression of myelin proteins or to anomalous myelination in the PFC and its connections. Clearly, the magnitude and extent of Cx43 and myelin changes detected after stress, though significant, are smaller than in connexin KO animals. However, during the last decade it has become clear that plasticity of myelin leading to significant, but not necessarily catastrophic, changes in the morphology or extent of myelin results in meaningful variations in function and connectivity (Nave, 2010; Nave and Ehrenreich, 2014; Fields, 2015; Fields et al., 2015).

### Involvement of Astrocytes in Neuroimmune Activation Elicited by Stress-Responses

Depression and stress responses are closely accompanied by dysregulation of immune activation and by expression of neuroimmune mediators produced by microglia and astrocytes (Hodes et al., 2015; Miller and Raison, 2016). Astrocytes carry receptors for various cytokines and chemokines and can thus respond to some of those mediators released by surrounding microglial cells or provided in the blood circulation (Maeng and Hong, 2019). Some of these responses involve chemokine expression that would in turn increase the ability to attract peripheral immune cells to the brain (Hennessy et al., 2015). Importantly, the actions of immune mediators on astrocytes directly influence their ability to perform essential functions (Sofroniew, 2014) in the support of neuronal activity, such as neurotransmitter metabolism (Muller and Schwarz, 2007), glutamate transport (Haroon et al., 2017, 2018; Piao et al., 2019), expression of aquaporins, maintenance of gap junctions (John et al., 1999; Karpuk et al., 2011) or expression of connexin 43 in a nuclear factor-kB (NF-kB)-dependent manner (Zhao et al., 2006). Conversely, connexin 43, which is present in astrocyte gap junctions interacting with the blood brain barrier (BBB), works as a main factor in regulating immune cell recruitment and antigen presentation at the BBB (Boulay et al., 2015). Thus, the sensitivity of astrocytes to inflammatory and other immune system-derived factors and the feedback they may exert to further regulate immune responses may be major contributors to neuronal metabolism and connectivity deficits that are thought to underpin the emotional and behavioral pathology caused by stress and other depressogenic neural mechanisms.

The intracellular molecular pathways involved in stress-induced activation of immune responses by astrocytes may involve the regulation of NF-kB by menin, because in non-stressed conditions this protein keeps in check the content of nervous system pro-inflammatory transcriptional activator NF-kB and the consequent downstream stimulation of interleukin 1ß (IL-1ß), while chronic stress results in menin reduction and subsequent activation of NF-kB and IL-1ß increase. Depression-like behaviors in the same animal model were prevented by the use of NF-kB inhibitors or IL1ß antagonists (Leng et al., 2018; Zhuang et al., 2018). Likewise, a polymorphism of gene *MEN1* (which encodes menin) was found to be significantly linked to higher risk for the onset of depression (Leng et al., 2018). In fact, the polymorphism reduces the binding of menin to NF-kB component p65, effectively enhancing the activation of Nf-kB and thus the production of IL1ß by astrocytes.

### Astrocyte Thrombospondin in Depression and Stress-Related Synaptic Alterations

Release of extracellular matrix proteins thrombospondin-1 and -2 by astrocytes is a strong promoter of synapse formation *in vitro* and *in vivo* (Christopherson et al., 2005; Jayakumar et al., 2014). Since changes of synaptic plasticity are involved in the pathophysiology of stress responses, depression, and other psychiatric disorders (Duman and Aghajanian, 2012; Yuen et al., 2012; Treccani et al., 2014; Csabai et al., 2018), some researchers have targeted the putative involvement of astrocyte-expressed thrombospondins 1 (TSP1) and 2 in the consequences of stress, including the generation of depression-like behaviors, as well as in the synaptic effects of antidepressant treatments. For instance, TSP-1 synthesis and mRNA expression are increased in the hippocampus after repeated electroconvulsive therapy, one of the most effective antidepressant treatments, although pharmacological antidepressants are apparently ineffective (Okada-Tsuchioka et al., 2014, 2017). In human subjects, plasma levels of TSP-1 levels were also found to be decreased in depression, at least in females (Okada-Tsuchioka et al., 2020).

### Astrocyte Processes and Aquaporin at the Blood Brain Barrier in Stress-Related Neuropathology

In the prefrontal cortex of human subjects with depression, the coverage of vessels by the aquaporin 4 (AQP-4)-immunoreactive endfeet of astrocytes is significantly reduced, even if the extent of those feet is not changed when labeled for the cytoskeletal protein GFAP (Rajkowska et al., 2013). Interestingly, in a genetic rat model for depression-like behaviors (Arauchi et al., 2018), AQP-4-positive astrocyte endfeet were also found to be reduced when compared with Wistar rats. Remarkably, electroconvulsive therapy was able to suppress depression-like behaviors and increased the extent of AQP-4 immunoreactive processes around blood vessels in the rat genetic model (Azis et al., 2019). In fact, the functions of astrocytes’ processes abutting at the basal lamina of arterioles are not limited to the control of BBB exchanges, but also include the regulation of the neurovascular coupling that depends on arteriole smooth muscle responses to extracellular potassium ions (Longden et al., 2014). This regulation may be significantly affected by elevated glucocorticoids in stress because corticoids, besides affecting smooth muscle potassium channels, would also alter coverage of arterioles by astrocytes processes, thus adding another risk factor for astrocyte-induced blood flow dysregulation upon the stress response.

Chronic stress can also cause astrocyte mediated alterations of cerebrovascular regulation through increases in oxidative damage elicited by neuroinflammation and oxidative stress. These processes are increased in the brain of subjects with depression, involving decreased levels of antioxidants and higher levels of products of lipid peroxidation or, in serum, accompanied by augmented amounts of malondialdehyde, decreased antioxidant enzymes such as glutathione peroxidase and catalase, and increased superoxide and nNOS (Burrage et al., 2018). Decreases in eNOS in astrocytes and endothelial cells after stress may displace the equilibrium between oxidative and antioxidant processes to favor excess generation of deleterious superoxide.

### Astrocyte Epigenetic Markers in Stress and Depression

As presented above, there are major effects of stress and probably other depressogenic mechanisms on the expression of astrocytes’ connexins 43 and 30 in brain cortical areas (Sun et al., 2012; Miguel-Hidalgo et al., 2014, 2018, 2019). These reductions also affect their corresponding mRNAs both in animal models of stress (Sun et al., 2012) and in humans subjects with depression (Nagy et al., 2016) and have been convincingly attributed to epigenetic marker alterations such as methylation of histone 3 at Cx43 and Cx30 genes in the prefrontal cortex using chromatin immunoprecipitation (Nagy et al., 2016). The epigenetic differences between subjects with depression and controls also extend to the pattern of DNA methylation for genes expressed at a high level in astrocytes as compared to other cell types (Nagy et al., 2015) including *GFAP, ALDHL1*, *SLC1A3, GJA1, GJB6*, *GLUL* and *SOX9*, all of which are found to be reduced in the dorsolateral prefrontal cortex of subjects with depression (Nagy et al., 2015). Depression-linked histone methylation modifications in astrocytes also have been detected for genes involved in the signaling of trophic factors such as *BDNF*, which is altered in some subjects with depression and in stress models. The levels of a BDNF receptor variant expressed in astrocytes, TrkB.T1, were found to be decreased in the prefrontal cortex of depressed suicide completers and inversely correlated with the levels of Hiistone 3 methylation at lysine 27 (Ernst et al., 2009a,b). It has been suggested that vulnerability to the effects of stress, at least of a social nature, could be related to the expression of the Trkb.T1 variant in astrocytes, because mice with higher expression levels of that variant express avoidance behaviors to a higher extent than control mice, behavior that would be related to the increased probability of epigenetic modifications at Trkb.T1 in astrocytes (Razzoli et al., 2011; Hodes et al., 2017).

Further research into the regulation of astrocyte glutamate uptake has also indicated that epigenetic alterations of glutamate transporter genes *EAAT1* and *EAAT2* (*GLAST* and *GLT-1* respectively in rats) may confer vulnerability to the effects of stress or the manifestation of depression.

Specificity of epigenetic changes in astrocytes as compared to changes in neurons is underscored by astrocyte-specific higher or lower expression of some of the enzymes that are in charge of executing epigenetic modifications, astrocytes featuring higher mRNA expression of histone deacetylase Hdac8, sirtuin Sirt8, histone methyl-transferase Mll3 or histone demethylase Kdm5d, while underexpressing histone deacetylase Hdac5, Hdac11 or histone demethylase Kdm2b (Zhang et al., 2014; Jakovcevski et al., 2016). This capability of astrocytes to differentially contribute to epigenetic changes may be of great relevance to the vulnerability to stress-related disorders and depression since disturbances in the expression of epigenetic enzymes and in the epigenetic changes themselves have been found to distinguish specific brain regions of subjects with depression as compared to non-psychiatric human subjects (Lopizzo et al., 2015; Jakovcevski et al., 2016). Other studies have pointed to factors involved in regulating the transcription of glutamate transporters such as the astrocyte-elevated gene which acts as corepressor of factor YY1 to repress the transcription of *GLT-1* and diminish glutamate uptake by astrocytes. YY1 also mediates repression of *GLAST* and *GLT-1* expression after exposure to inflammatory cytokine tumor necrosis factor α (TNF-α) by recruiting histone deacetylases to the promoter of glutamate transporter genes (Pajarillo et al., 2019). Some of the changes in epigenetic regulation of glutamate transporter expression may also be involved in depression-like behaviors and cognitive alterations secondary to neurological conditions, such as subarachnoid hemorrhage, in which astrocyte histone deacetylase 2 negatively regulates the expression of *GLT-1* (Tao et al., 2020).

### Effects of Early Stress Exposure on Astrocytes and Their Role in the Development of Depression

Paramount among the studies addressing the vulnerability to depressive and anxiety symptoms are those focusing on the effects of early life adversity and stress on the probability of developing depressive and other psychiatric illnesses during and adolescence and adulthood (Chen and Baram, 2016). Various of these studies have been based on different forms of early adversity, including maternal separation/deprivation, malnutrition, overnutrition or immune challenges. A recent review has stressed the importance of early adversity in the responses of astrocytes to immune challenges later in life, as well the effects of disturbed astrocyte metabolism due to malnutrition, which could set the stage for astrocyte priming as a factor for the level of vulnerability to affective and other psychiatric disorders (Abbink et al., 2019). In the hippocampus, the multifaceted physiological roles of astrocytes and their involvement in functional plasticity such as long-term potentiation have been recently reviewed by Çalişkan et al. (2020), also indicating that early or juvenile stress results in reduced GFAP expression and diminished complexity of morphological features in astrocytes as well as reduced uptake of glutamate and GABA, and impaired conversion of glutamate to glutamine (Çalişkan et al., 2020). Repeated or chronic maternal separation in infant rats during the lactating period was also found to cause drastic reductions in the number of GFAP-immunoreactive astrocytes in the prefrontal cortex, hippocampus and striatum (Banqueri et al., 2019). For instance, maternal separation stress results in depletion of GFAP and S100b immunoreactive astrocytes in the medial precentral cortex of the Wistar rat strain (Musholt et al., 2009). Similarly, in Fischer rats, which are constitutively highly responsive to stress, 14 days of early maternal deprivation leads to reduced density of GFAP-immunoreactive astrocytes in the prefrontal cortex, hippocampal regions and the amygdala (Leventopoulos et al., 2007) in adult animals. In guinea pigs, antenatal stress of dams results as well in reduced GFAP immunostaining of CA1 hippocampal astrocytes when examined in 21-days old offspring (Bennett et al., 2015). In view of the lower immunoreactivity of GFAP immunoreactive astrocytes and the lower levels of GFAP observed in the prefrontal cortex in postmortem human brains from younger subjects with major depression (as compared to older subjects or to non-psychiatric controls) (Miguel-Hidalgo et al., 2000; Si et al., 2004), early stress, adversity or excessive immune activation may prime astrocytes to facilitate, at least in some human subjects, manifestations of depression in late-adolescence and adulthood.

Since early life stress (ELS) also involves the activation of hormonal responses regulated by the hypothalamic-pituitary-adrenal axis, some research has addressed cellular and neurotransmitter dysfunction in the hypothalamus and a putative involvement of astrocytes. In mature hypothalamic neurons, the ability of neurosteroids to reduce overactivation was found to be curtailed by ELS. This effect is dependent on an excessive glutamatergic activation that derives in part from impaired astrocytic regulation of extracellular glutamate levels (Gunn et al., 2013). This alteration was concomitant with morphological changes of GFAP-immunoreactive astrocyte processes and a reduction of the immunoreactivity of astrocytic glutamate transporters (Gunn et al., 2013).

Long-lasting astrocyte glutamate transport deficits may lead to dysfunction of specific circuits known to be involved in the pathophysiology of affective and psychiatric disorders. For instance, in the medial prefrontal cortex of rodents, astrocytic glutamate transport activity plays a major role in modulating the connections between the prefrontal cortex and the serotonergic neurons of the raphe nucleus (Fullana et al., 2020). Accordingly, prolonged suppression of the astrocyte-dependent glutamate transport capacity in the prefrontal cortex of mice results in depression-like behaviors (Fullana et al., 2019a,b), although the effects of acute vs chronic stress on the connections between the various subdivisions of the PFC and the serotonergic raphe neurons remain to be fully elucidated (Fullana et al., 2020).

## Conclusion

**FIGURE 1:**
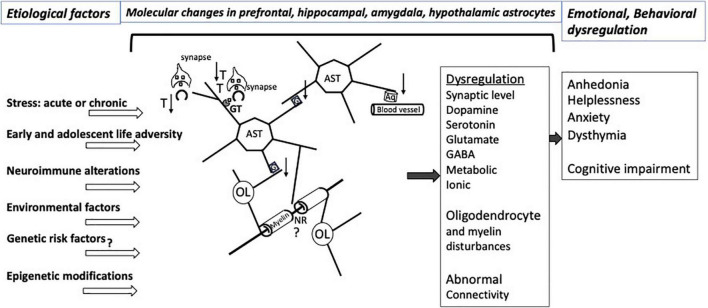
Illustration of relevant interactions involving CNS astrocytes that would be affected by various types of stress and other factors predisposing to affective disorders. The pathological interactions with astrocytes result in a variety of molecular and neurotransmitters alterations in the prefrontal cortex, hippocampus, amygdala, striatum, hypothalamus involving dysregulation of at least dopamine, serotonin, glutamate and GABA neurotransmission, with varying degrees of regional specificity. Stress exposure and other risk factors for neuropathology itself also may lead to pathological disturbances of astrocyte-contributions to BBB regulation and to the extracellular matrix NRs and other parts of the white matter. More recent research work has revealed epigenetic abnormalities or changed levels of specific miRNAs and other non-coding RNAs in affective disorders that may contribute to specific emotional and cognitive symptoms in particular brain regions. Nonetheless, substantial additional research is required to improve our understanding of mechanisms underpinning the relationship of cellular and molecular pathology to epigenetic and non-coding RNA markers that result in dysfunctional astrocyte contacts with neurons and oligodendrocytes, eventually leading to abnormal brain connectivity in affective disorders. Aq, aquaporin 4; AST, astrocytes; G, gap junctions; GT, glutamate and GABA transporters; NR, node of Ranvier; OL, oligodendrocyte; T, thrombospondin; ↑, up-regulation; ↓, down-regulation. At the synapse, squares represent neurotransmitters.

Astrocytes’ multifaceted involvement in various aspects of neuronal metabolism, support of neurotransmission, supply of neurotrophic factors, neuroimmune reactivity, myelin maintenance, and regulation of signal propagation as well as their capacity to directly respond to hormonal and other molecular adaptations caused by stress sets them to be major players in determining the behavioral and emotional vulnerability to the effects of stress (Figure 1). These effects may be long lasting because at different stages of development and in adulthood there is substantial epigenetic regulation of gene expression in astrocytes, and the astrocytes themselves are crucial in supporting the development of other neural cells, synaptic connections, and axonal pathways in the central nervous system. Some of the epigenetic and molecular mechanisms regarding glutamate transport, gap junction communication and neuroimmune regulation that are related to stress effects are starting to be ascertained. However, much remains to be learned to understand the specific role of those and other modifications in the astroglial contribution to the vulnerability to and maintenance of stress-related disorders and depression, and for leveraging that knowledge to achieve more effective psychiatric therapies.

## Author Contributions

The author confirms being the sole contributor of this work and approved it for publication.

## Conflict of Interest

The author declares that the research was conducted in the absence of any commercial or financial relationships that could be construed as a potential conflict of interest.

## Publisher’s Note

All claims expressed in this article are solely those of the authors and do not necessarily represent those of their affiliated organizations, or those of the publisher, the editors and the reviewers. Any product that may be evaluated in this article, or claim that may be made by its manufacturer, is not guaranteed or endorsed by the publisher.
